# Supplementary material for: The Role of mpMRI in the Assessment of Prostate Cancer Recurrence Using the PI-RR System: Diagnostic Accuracy and Interobserver Agreement in Readers with Different Expertise
Source: Diagnostics (Basel). 2023 Jan 20;13(3):387. doi: 10.3390/diagnostics13030387 (PMC9914595; doi:10.3390/diagnostics13030387)
Supplement: Supplementary file 1 [file diagnostics-13-00387-s001.zip › diagnostics-2128318-supplementary.pdf]

Supplementary material

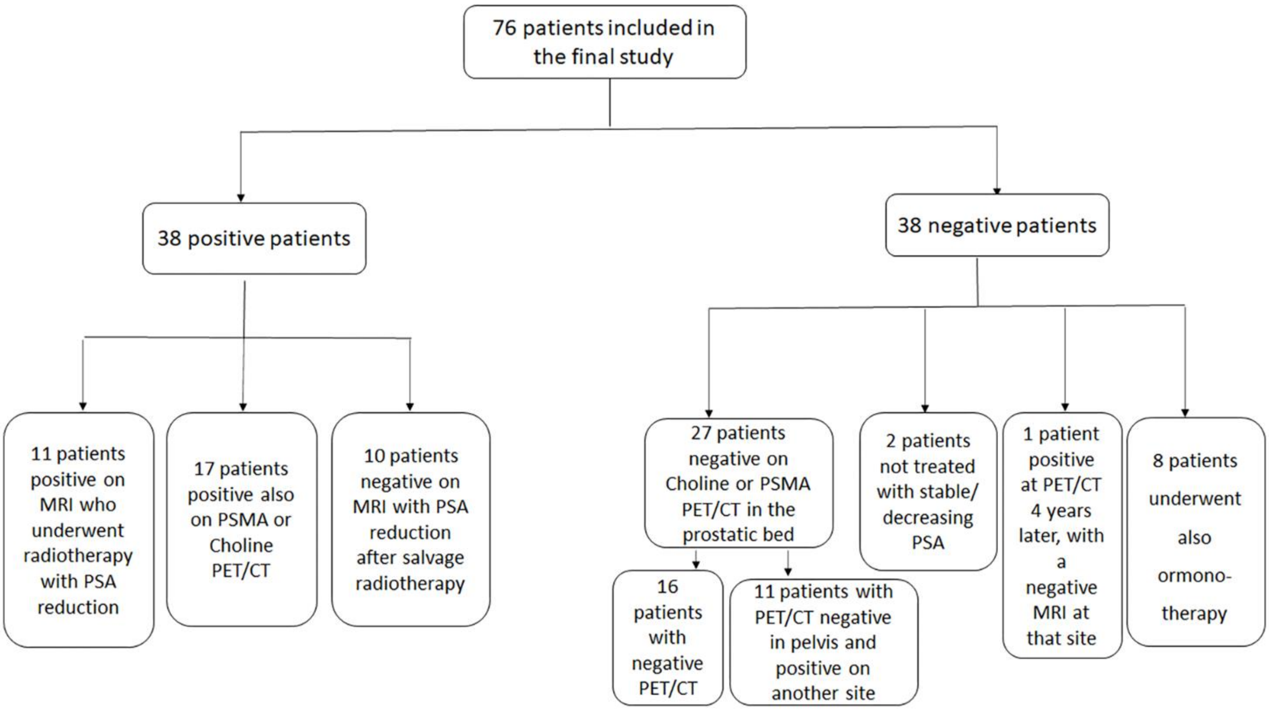

Supplementary Figure S1 - description of patients included in the final study

Of the 38 true positive patients, 11 had a positive MRI and underwent a therapy with decrease of the PSA levels, 17 had a choline or PSMA PET positive in the same area where the mpMRI found out a suspicious area; 10 patients had a negative MRI, but their PSA values decreased after the early salvage therapy. Of the 38 true negative patients, 16 patients were negative even at the PET CT scan, 11 patients had a positive PET CT at another location (1 patient had a bone metastasis and 10 patients had lymph nodal metastases), 1 patient was positive at the PET CT follow up, four years later but the MRI was negative in that site even on a retrospective imaging review; 2 patients had not been treated and had a stable o decreasing PSA values; 8 patients needed androgen deprivation therapy after salvage radiotherapy to decrease PSA.

Sequences	TR(ms)	TE(ms)	B value	Slice Thickness (mm)	Scan time(min sec)	Temporal resolution
T2W TSE Ax	5760	110		3(no gap)		
T2W TSE Cor e Sag	6970	110		3(no gap)		
T1W Ax	575	20		5 (no gap)		
DWI-EPI Ax	5800	68	1500	3(no gap)		
DWI-EPI Ax<br>(used for ADC map)	5200	63	50-750-1000	3(no gap)		
DCE	4,6	1,76		3(no gap)	2,26 min	9 sec

Supplementary Table S1

Acquisition parameters for multiparametric magnetic resonance imaging of the prostate.

	Reader 1	Reader 2	Reader 3
**Sens**	68.4 (51.3-82.5)	64.5 (45.4-80.8)	70.3 (53-84.1)
**Spec**	93.9 (79.8-99.3)	100 (89.7-100)	89.2 (74.6-97)
**AUC**	0.81 (0.73-0.90)	0.82 (0.74-0.91)	0.79 (0.70-0.88)
**PPV**	92.9 (76.5-99.1)	100 (83.2-100)	86.7 (69.3-96.2)
**NPV**	72.1 (56.3-84.7)	75.6 (60.5-87.1)	75 (59.7-86.8)

Supplementary Table S2

Diagnostic Performance for Each Reader for Recurrence Detection without the score of uncertainty

The percentage of PI-RR score 3 (score of uncertainty) was 6.6% for reader 1, 14.5% for reader 2 and 2.6% for reader 3.

**<u>PSA below</u>**			
	**Reader 1**	**Reader 2**	**Reader 3**
**Sens**	55.0 (31.5-76.9)	55.0 (31.5-76.9)	60.0 (36.1-80.9)
**Spec**	77.8 (57.7-91.4)	88.9 (70.8-97.6)	81.5 (61.9-93.7)
**AUC**	0.66 (0.53-0.80)	0.72 (0.59-0.84)	0.71 (0.57-0.84)
**PPV**	64.7 (38.3-85.8)	78.6 (49.2-95.3)	70.6 (44.0-89.7)
**NPV**	70.0 (50.6-85.3)	72.7 (54.5-86.7)	73.3 (54.1-87.7)

**<u>PSA above</u>**			
	**Reader 1**	**Reader 2**	**Reader 3**
**Sens**	71.4 (29-96.3)	71.4 (29.0-96.3)	57.1 (18.4-90.1)
**Spec**	100 (15.8-100)	100 (15.8-100)	100 (15.8-100)
**AUC**	0.86 (0.68-1.00)	0.86 (0.68-1.00)	0.79 (0.59-0.98)
**PPV**	100 (47.8-100)	100 (47.8-100)	100 (39.8-100)
**NPV**	50 (6.8-93.2)	50 (6.76-93.2)	40 (5.3-85.3)

Supplementary Table S3

Diagnostic Performance for Each Reader for Recurrence Detection in patients who underwent radical prostatectomy with a PSA level below 1ng/ml or above it.

In the setting of BCR with patients previously treated with radical prostatectomy, the accuracy of MRI is slightly lower when PSA level is below 1 ng/ml.
